# Intracellular arginine-dependent translation sensor reveals the dynamics of arginine starvation response and resistance in ASS1-negative cells

**DOI:** 10.1186/s40170-021-00238-9

**Published:** 2021-01-21

**Authors:** Leonard C. Rogers, Jing Zhou, Adriana Baker, Charles R. Schutt, Prashanta K. Panda, Brian A. Van Tine

**Affiliations:** 1 Division of Medical Oncology, Washington University in St. Louis, St. Louis, Missouri 63110 USA; 2 The First Affiliated Hospital of Nanchang University, Nanchang, 330006 Jiangxi China; 3 University of Nevada, Las Vegas, Las Vegas, NV 89154 USA; 4 Division of Pediatric Hematology/Oncology, St. Louis Children’s Hospital, St. Louis, MO 63110 USA; 5 Siteman Cancer Center, St. Louis, MO 63110 USA

**Keywords:** Arginine, Sensor, Biosensor, Sarcoma, Arginine deiminase, Argininosuccinate synthetase 1, ASS1, Tumor heterogeneity

## Abstract

**Background:**

Many cancers silence the metabolic enzyme argininosuccinate synthetase 1 (ASS1), the rate-limiting enzyme for arginine biosynthesis within the urea cycle. Consequently, ASS1-negative cells are susceptible to depletion of extracellular arginine by PEGylated arginine deiminase (ADI-PEG20), an agent currently being developed in clinical trials. As the primary mechanism of resistance to arginine depletion is re-expression of ASS1, we sought a tool to understand the temporal emergence of the resistance phenotype at the single-cell level.

**Methods:**

A real-time, single-cell florescence biosensor was developed to monitor arginine-dependent protein translation. The versatile, protein-based sensor provides temporal information about the metabolic adaptation of cells, as it is able to quantify and track individual cells over time.

**Results:**

Every ASS1-deficient cell analyzed was found to respond to arginine deprivation by decreased expression of the sensor, indicating an absence of resistance in the naïve cell population. However, the temporal recovery and emergence of resistance varied widely amongst cells, suggesting a heterogeneous metabolic response. The sensor also enabled determination of a minimal arginine concentration required for its optimal translation.

**Conclusions:**

The translation-dependent sensor developed here is able to accurately track the development of resistance in ASS1-deficient cells treated with ADI-PEG20. Its ability to track single cells over time allowed the determination that resistance is not present in the naïve population, as well as elucidating the heterogeneity of the timing and extent of resistance. This tool represents a useful advance in the study of arginine deprivation, while its design has potential to be adapted to other amino acids.

**Supplementary Information:**

The online version contains supplementary material available at 10.1186/s40170-021-00238-9.

## Background

Arginine is acquired either extracellularly from the blood stream or synthesized intracellularly by enzymes from the urea cycle. The rate-limiting step in arginine biosynthesis is catalyzed by argininosuccinate synthetase 1 (ASS1) (EC 6.3.4.5) [1]. As most arginine is made and exported from the kidney cells for utilization by other cell types, many cancers take advantage of this extracellular supply by silencing ASS1 expression without compromising their arginine supply [2–6]. Cancer is thought to silence ASS1 to free aspartate pools for the biosynthesis that is associated with rapid cell division [7].

ASS1-deficient cells must import extracellular arginine to survive and grow, making arginine depletion an attractive therapeutic strategy. Therefore, multiple arginine-depleting enzymes have been developed. The most clinically tested of these is arginine deiminase conjugated to polyethylene glycol, ADI-PEG20 [5, 8]. This enzyme functions extracellularly to break down arginine into citrulline and ammonia [9]. Most cells in the body are not greatly affected, as their ASS1 expression levels are sufficient for arginine production and/or their metabolic requirements for arginine are much lower than rapidly proliferating cells.

There are multiple possible mechanisms of resistance to arginine starvation, but the most common mechanism is the re-expression of ASS1 [5, 10–12]. This is because the gene remains unaltered at the sequence level in most ASS1-deficient cells, as it is silenced by methylation [13, 14]. Mechanistically, when arginine is depleted by ADI-PEG20, c-Myc is activated and translocates to the nucleus, where it binds to the *ASS1* promoter and upregulates transcription, leading to intracellular arginine production [10, 11, 15, 16]. The timing and heterogeneity of the emergence of resistance are important to understand for clinical translation, but until now, tools have been lacking to effectively study arginine metabolism at the single-cell level.

Many methods have been developed to measure biological arginine concentrations [17]. While these assays can be useful, they are often expensive and difficult to use, and most are aimed toward food and medical systems [17]. Very few of the available methods measure intracellular concentrations in live cells, and most of these do not allow for real-time measurements [17]. The only published dynamic intracellular sensor of arginine in mammalian cells relies on an arginine-binding protein from *Chlamydia pneumoniae* [18]. Unfortunately, this system is not suitable for studying biologically relevant arginine concentrations, as it responds only to arginine concentrations that are higher than physiologic arginine levels [18].

Most organisms also have mechanisms for sensing amino acid concentrations. In higher organisms, the mTOR pathway senses nutrients, including arginine [19–21], and is tied to cell proliferation [22]. In bacteria, *trp* operon attenuation utilizes a potentially useful mechanism. As the operon is transcribed, a ribosome translates the nascent mRNA, following closely behind the RNA polymerase. When tryptophan is abundant, the ribosome proceeds along the mRNA, allowing a transcription termination signal to form in the mRNA and halt transcription [23]. When tryptophan is scarce, the ribosome stalls, an alternate mRNA structure forms, and transcription continues [23]. Such ribosomal stalling during amino acid scarcity has been demonstrated in human cells specifically in response to arginine deprivation [24].

This study characterizes and describes the development of an arginine translation sensor (ArgSen) based on the principle of ribosomal stalling. Several different structural components proved useful in studying cellular responses to arginine deprivation, as they were combined to create a novel chimeric protein that would need to satisfy multiple criteria. The ArgSen monitors cellular arginine-dependent translation in individual cells and entire populations over time without adversely affecting growth or response to stimuli. We demonstrate that when ASS1-deficient cells are treated with ADI-PEG20, there is a homogeneous decrease in translational capacity, but a heterogeneous pattern of resistance. Finally, we calculate the minimum concentration of arginine needed for optimal sensor translation.

## Methods

### Cell culture

SKLMS1, SKUT1, and SKMEL2 cell lines were obtained from the American Type Culture Collection (Manassas, VA) and are listed in Additional file 1. WT cancer cell lines were grown in MEM (Thermo Fisher Scientific, Waltham, MA) supplemented with 10% FBS (Bio-Techne, Minneapolis, MN), 1.3% 100× penicillin-streptomycin (10,000 U/mL) (Thermo Fisher Scientific), and 2.5 μg/mL Plasmocin (InvivoGen, San Diego, CA). LTAT cells were grown in this medium with 1 μg/mL ADI-PEG20 (Polaris, San Diego, CA) added. All ADI-PEG20 treatments were performed by replacing media with media that had been pre-treated with 1 μg/mL ADI-PEG20 for at least 8 h at 37 °C.

Mouse embryonic fibroblasts (MEFs) were generated as detailed later. MEFs were grown in IMDM (Thermo Fisher Scientific) supplemented with 20% FBS, 1% 100× MEM non-essential amino acids (Thermo Fisher Scientific), 0.0007% 2-mercaptoethanol (MilliporeSigma, Burlington, MA), 1% 100× penicillin-streptomycin (10,000 U/mL), and 2.5 μg/mL Plasmocin.

### Automated cell imaging

Cells were transduced with IncuCyte® NucLight Red Lentivirus Reagent (Essen BioScience, Ann Arbor, MI) and selected with puromycin. SKLMS1, SKUT1, and SKMEL2 cells were plated in 96-well plates at 3 × 10^3^, 7.5 × 10^3^, and 5 × 10^3^ cells per well, respectively. The next day, fresh phenol red-free medium was added to start treatment along with 50 nM YOYO™-1 Iodide (Thermo Fisher Scientific), which fluorescence green within the nuclei of dead cells. Images were taken with IncuCyte® ZOOM or IncuCyte® S3 (Essen BioScience) automated fluorescent microscopes at a rate of 0.5–1 per hour. Red and green cell numbers were quantified automatically with an analysis algorithm within the IncuCyte software (Essen BioScience).

SKLMS1, SKUT1, and SKMEL2 cells expressing the indicated GFP arginine sensor variants were plated in 96-well plates at 3 × 10^3^, 7.5 × × 10^3^, and 5 × 10^3^ cells per well, respectively. The next day, fresh phenol red-free medium was added to start treatment. Glutamine deprivation experiments used media supplemented with One Shot™ dialyzed FBS (Thermo Fisher Scientific) in place of standard FBS, and 2 mM L-glutamine (Corning, Corning, NY) was either added or omitted. Images were taken with IncuCyte® ZOOM or IncuCyte® S3 automated fluorescent microscopes at a rate of 0.5–1 per hour. Sensor fluorescence was quantified automatically with an analysis algorithm within the IncuCyte software, and average integrated intensities of green fluorescence in individual cells were taken.

SILAC RPMI 1640 Flex Media, with no glucose and no phenol red (Thermo Fisher Scientific) was used in experiments to vary arginine concentrations. Glucose (Agilent Technologies, Santa Clara, CA), l-glutamine, and l-lysine hydrochloride (MilliporeSigma) were added to the same concentrations as RPMI 1640 (Thermo Fisher Scientific), along with 10% dialyzed FBS and 1.3% pen/strep. l-arginine (MilliporeSigma) was added to the indicated concentrations. Cells were grown and passaged in RPMI 1640 for at least 1 week before arginine concentration experiments. Fluorescence of the arginine sensor was measured with the IncuCyte S3 at 0, 1, and 2 h of the indicated treatments. The areas under the curve for this time period were then plotted against arginine concentration, with 0 represented as 0.01 μM. Data were fit to the following equation:

$$ Y=\mathrm{Bottom}+\frac{\left({X}^{HillSlope}\right)\times \left( Top- Bottom\right)}{\left({X}^{HillSlope}+ EC5{0}^{HillSlope}\right)} $$

Effective concentration (EC) of any other value (F) was then calculated with the following equation:

$$ \mathrm{ECF}={\left(\frac{F}{100-F}\right)}^{\sqrt{HillSlope}}\times EC50 $$

where indicated, 100 μM cycloheximide (CHX) (MilliporeSigma) or 1 μM bortezomib (BTZ) (Millennium Pharmaceuticals, Cambridge, MA) was added to media. Fluorescence data in the presence of CHX were fit to the following exponential one-phase decay equation:

$$ Y=\left({Y}_0- Plateau\right)\ast {e}^{-K\ast X}+ Plateau $$

Half-lives were calculated as (ln(2)/K).

### Individual cell tracking

Arginine sensor fluorescence of individual cells was tracked over time by hand within the IncuCyte S3 software by recording the integrated intensity of green objects (nuclei) detected by the analysis algorithm. One hundred cells were initially targeted for tracking in each experimental condition. Results include data from less than 100 cells, as not all cells were able to be tracked with certainty. At timepoints where fluorescence in a cell was too low to be detected by the algorithm, the integrated intensity value was recorded as 0.

### Capillary electrophoresis

For obtaining lysates after a time course of ADI-PEG20, SKLMS1 cells were plated in 60-mm dishes, and treatments were administered so that all ended simultaneously. 7.5 × 10^4^ LTAT cells were plated for every timepoint. 7.5 × 10^4^ WT cells were plated for samples with 12 or fewer hours of treatment, and the number was doubled for each full day of treatment, with 6 × 10^5^ cells plated for 72 h. Treatment was started by the addition of fresh media the next day. All samples received fresh media 2 h before harvesting. After lysis, immunoblots were performed with a Wes automated immunoblot machine (Bio-Techne) according to the manufacturer’s protocol [25]. Each sample was normalized to its own total protein [25]. Two ASS1 primary antibodies were used: a non-commercial mouse monoclonal from Polaris and a rabbit polyclonal ab175607 (Abcam, Cambridge, MA). Mouse monoclonal GFP antibody sc-9996 (Santa Cruz Biotechnology, Dallas, TX) was used to detect the arginine sensor. Two bands were detected at approximately the expected molecular size of the GFP sensor, and both were quantified and added together before normalization to total protein. Although a single band was expected, the identity of the two separate bands was not investigated. Antibodies are listed in Additional file 2.

### Cloning

A gBlocks® Gene Fragment (Integrated DNA Technologies, Coralville, IA) of the first arginine sensor variant (ArgSen (-) NLS), containing a P2A site and Fast-FT without a nuclear localization sequence (NLS), was cloned using AscI and NotI restriction sites. All gBlocks® in this study were ordered with extra nucleotides on each end to allow for direct restriction digestion and ligation into the vector. All arginine sensor variations were first cloned into plasmid pKLV2-EF1a-BsdCas9-W (replacing the original insert), then subcloned into plasmid pLV-EF1a-IRES-Puro. pKLV2-EF1a-BsdCas9-W was a gift from Kosuke Yusa (Addgene plasmid # 67978 ; http://n2t.net/addgene:67978 ; RRID:Addgene_67978) [26]. pLV-EF1a-IRES-Puro was a gift from Tobias Meyer (Addgene plasmid # 85132; http://n2t.net/addgene:85132; RRID:Addgene_85132) [27]. Successful cloning was confirmed by Sanger sequencing with primers EF1a fwd, pKLV2 seq rev, pLV seq rev, and Rad23b end fwd. All oligonucleotides in this study were ordered from Integrated DNA Technologies and are listed in Additional file 3.

After cloning ArgSen (-) NLS, multiple modifications were made. A C-terminal SV40 Large T-antigen NLS was added by amplification of Fast-FT with SalI BamHI FastFT fwd and NotI NLS FastFT rev primers, followed by replacement of Fast-FT with Fast-FT-NLS using BamHI and NotI restriction sites (ArgSen). Separately, the P2A site was removed by SalI restriction digestion followed by ligation (Argsen (-)P2A (-)NLS). Fast-FT in ArgSen (-)P2A (-)NLS was then replaced by enhanced green fluorescent protein with a C-terminal NLS (GFP-NLS) that had been PCR amplified with SalI BamHI GFP fwd and NotI NLS GFP rev primers, using BamHI and NotI restriction sites (GFP ArgSen). The polyarginine region in this variant was then replaced with a region of identical length encoding random non-arginine amino acids, obtained as a gBlock® and cloned with restriction sites XbaI and EcoRI (GFP RanSen).

Variations of the sensor with regions deleted to test degradation were derived from GFP ArgSen. DNA oligonucleotide pairs designed to replace targeted regions with short linkers were phosphorylated on their 5’ ends by T4 Polynucleotide Kinase (New England Biolabs, Ipswich, MA), then annealed. After annealing, each double-stranded oligonucleotide possessed complementary overhangs with the correct sequences to bind to the digested restriction sites on either side of its target region. The target region was cut out of the vector at these sites, and the newly annealed oligonucleotide was ligated in. NucGFP was made with Reporter only oligonucleotides using AscI and BamHI restriction sites. Δ Degradation Domain was made with Proteasomal del oligonucleotides using AscI and BstBI restriction sites. Δ Disordered Region was made with Disordered del oligonucleotides using AscI and NsiI restriction sites. Δ Rad23b UbL was made with Rad23b del oligonucleotides using NsiI and XbaI restriction sites. After publication, all constructs will be available from Addgene (Watertown, MA).

Lentiviral particles were made with Lenti-X™ 293 T cells (Takara Bio USA, Mountain View, CA). Experimental cells were transduced with virus and selected with puromycin.

### Flow cytometry

SKLMS1 ArgSen cells were plated in 6-well plates for a time course of ADI-PEG20, along with control WT cells for each timepoint. To enable simultaneous harvesting at similar levels of confluency, the following numbers of cells were plated per well according to the length of ADI-PEG20 treatment: 1 × 10^5^ for 72 h, 6.5 × 10^4^ for 48 h, 4 × 10^4^ for 24 and 18 h, and 2.5 × 10^4^ for 12 or fewer hours. All samples received fresh media 2 h before harvesting. Cells were washed with PBS, harvested with trypsin, and measured for fluorescence of blue Fast-FT by flow cytometry with a 407-nm laser and 50-nm wide bandpass filter centered at 450 nm. Fluorescence values of paired WT samples were subtracted from ArgSen samples, and values were then normalized to untreated fluorescence levels.

### RT-qPCR

For isolating RNA after a time course of ADI-PEG20 treatment, SKLMS1 cells were plated in 60-mm dishes, and treatments were administered so that all ended simultaneously. 7.5 × 10^4^ cells were plated for samples with 12 or fewer hours of treatment, and double for each full day of treatment, with 6 × 10^5^ cells plated for 72 h. All samples received fresh media 2 h before harvesting. Cells were washed in ice-cold PBS, scraped, and centrifuged. RNA was isolated by Direct-zol™ RNA Miniprep Plus kit (Zymo Research, Irvine, CA) according to the manufacturer’s protocol. The High-Capacity cDNA Reverse Transcription Kit (Thermo Fisher Scientific) was used to produce cDNA according to the manufacturer’s protocol. The *Power* SYBR™ Green PCR Master Mix (Thermo Fisher Scientific) was then used to quantify arginine sensor and GAPDH mRNA according to the manufacturer’s protocol. qrtPCR ArgSen fwd, qrtPCR ArgSen rev, qrtPCR GAPDH fwd, and qrtPCR GAPDH rev primers were used in the RT-qPCR reactions.

### Protein translation assay

SKLMS1 WT cells were plated at 6 × 10^3^ cells per well in a 96-well plate. The next day, media was replaced and indicated treatments were given at indicated times so that the assay began simultaneously for all samples. The Click-&-Go Plus 647 OPP Protein Synthesis Assay Kit (Click Chemistry Tools, Scottsdale, AZ) was used according to manufacturer’s instructions. Red fluorescence was measured on the IncuCyte S3, and cell-by-cell analysis was performed to quantify the mean intensity of fluorescence in each cell. The average fluorescences of CHX controls for each timepoint were subtracted from each sample before normalization to the untreated 0 timepoint.

### Mouse embryonic fibroblast generation

HEPD0731_5_F08 mutant embryonic stem cell clones were ordered from the European Conditional Mouse Mutagenesis Program (EUCOMM). These cells contain an allele of *ASS1* harboring FRT-flanked lacZ and neomycin resistance genes, followed by a loxP site upstream of critical exon 4 and another loxP site downstream of exon 4. Embryos were grown from these stem cells, and the resulting mice were crossed with mice expressing FLP recombinase. This cross removed the cassette containing lacZ and neomycin resistance genes, allowing the *ASS1* allele to encode functional ASS1 protein. These mice were then backcrossed to C57BL/6 mice with confirmed FLP-negative genotypes three times to remove FLP and possess *ASS1*^F/+^ alleles. ASS1^F/+^ mice were crossed to each other, and a colony of ASS1^F/F^ mice was established.

MEFs were generated from embryos harvested from an *ASS1*^F/F^ × *ASS1*^F/F^ mating. MEFs were allowed to grow normally and became spontaneously immortalized. MEFs were infected with either Ad5CMVCre or Ad5CMVcytoLacZ adenoviral particles (University of Iowa, Iowa City, IA) to knock out *ASS1* or serve as a negative control, respectively. Complete knockout of *ASS1* was confirmed by both genotyping and immunoblot.

### Metabolomics

For metabolomic analysis, SKLMS1 WT and LTAT cells expressing GFP ArgSen were plated in replicates of four on 100 mm dishes at 2 × 10^6^ cells per dish for every condition except LTAT with 48 h of ADI-PEG20, which had 1 × 10^6^ cells. The next day, cells were treated with ADI-PEG20 for the indicated time periods, with each sample getting fresh media 1 h before harvesting. Viable cells were counted for one sample from each condition, to be used for normalization. Metabolites were extracted from the remaining three samples with methanol using the protocol for extraction of metabolites from adherent cells from Human Metabolome Technologies America (HMT) (Boston, MA). Purified metabolites and viable cell counts were then sent to HMT for metabolomic analysis. Data from HMT were then normalized to ratios of individual sample total metabolites to average total metabolite levels per 1 × 10^6^ cells. The concentrations of arginine in ADI-PEG20-treated MEM and untreated MEM were also determined by HMT.

### Cell volume determination

For determining cell volume after 48 h of ADI-PEG20 treatment, cells were plated and treated identically to the metabolomics experiment, then harvested by trypsin. Samples were mixed well, and 20 μL of each were taken, mixed with an equal volume of trypan blue (Thermo Fisher Scientific), pipetted into Countess™ Cell Counting Chamber Slides (Thermo Fisher Scientific), and analyzed by a Countess™ II Automated Cell Counter (Thermo Fisher Scientific). Average cell diameter was calculated from this data. The remaining cells were analyzed by flow cytometry, measuring forward scatter intensity to determine relative cell diameters. Individual and average cell volumes were then calculated with FlowJo software (FlowJo, Ashland, OR).

### Statistics

Data were analyzed using GraphPad Prism 8 Software (GraphPad Software, San Diego, CA). mRNA and GFP immunoblot error bars represent standard error of the mean. All other error bars represent standard deviation. ASS1 protein expression differences were analyzed by unpaired *t* test. Growth rate differences were analyzed by one-way ANOVA. Metabolite differences at 48 h were analyzed by Welch’s *t* test. “ns” denotes non-significant difference between groups. * denotes *p* < .05, ** denotes *p* < .01, *** denotes *p* < .001, and **** denotes *p* < .0001.

## Results

### Resistance to ADI-PEG20

**Fig. 1:**
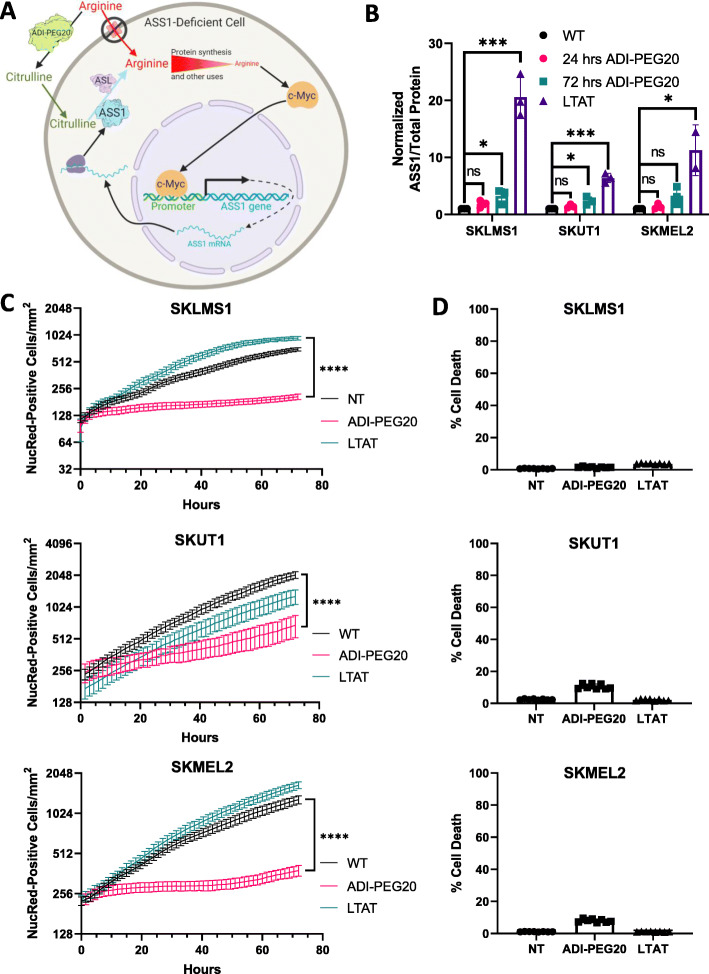
Response to arginine deprivation in ASS1-deficient cells**. a** Schematic overview of ADI-PEG20 treatment and response in ASS1-deficient cells. **b** ASS1 expression with ADI-PEG20 treatment. **c** Growth of cells with ADI-PEG20 treatment. **d** Cell death at 72 h of ADI-PEG20 treatment. All error bars represent standard deviation

The established working model of resistance to arginine starvation is demonstrated in Fig. 1a [10–12]. In ASS1-deficient cells, arginine is imported from the extracellular space. Upon the addition of ADI-PEG20, extracellular arginine is converted to citrulline, which causes a cellular starvation state that induces a c-Myc-dependent re-expression of ASS1 [10–12]. This is the primary mechanism of arginine starvation resistance, as re-expression of ASS1 allows the intracellular production of arginine through urea cycle enzymes. As more ASS1 is expressed, the cell can synthesize more arginine from citrulline, thus allowing cells to grow in the absence of extracellular arginine. ASS1-deficient cells moderately increase expression of ASS1 in the short term in response to arginine deprivation, but in the long term, ASS1 is greatly upregulated (Fig. 1b) [10–12].

To model the response of cells to the arginine starvation state, two well-characterized human sarcoma cell lines, SKLMS1 and SKUT1, and one human melanoma cell line, SKMEL2, were used [12, 16, 28]. As these lines express very little ASS1 at baseline, long-term ADI-PEG20 treatment (LTAT) resistant cell lines were derived as previously described by Kremer et al. [12]. The longer the cell lines were cultured with ADI-PEG20, and the more ASS1 protein was expressed (Fig. 1b). Next, we confirmed that arginine starvation in ASS1-negative cell lines leads to a cytostatic response, as all three WT cell lines demonstrated significantly decreased cellular proliferation when monitored by IncuCyte NucLight Red counts over 72 h, whereas LTAT cells grew at a similar rate as untreated WT cells (Fig. 1c). Finally, we demonstrated that the cell death is not meaningfully increased in the arginine starvation state (Fig. 1d).

### Arginine sensor design

**Fig. 2:**
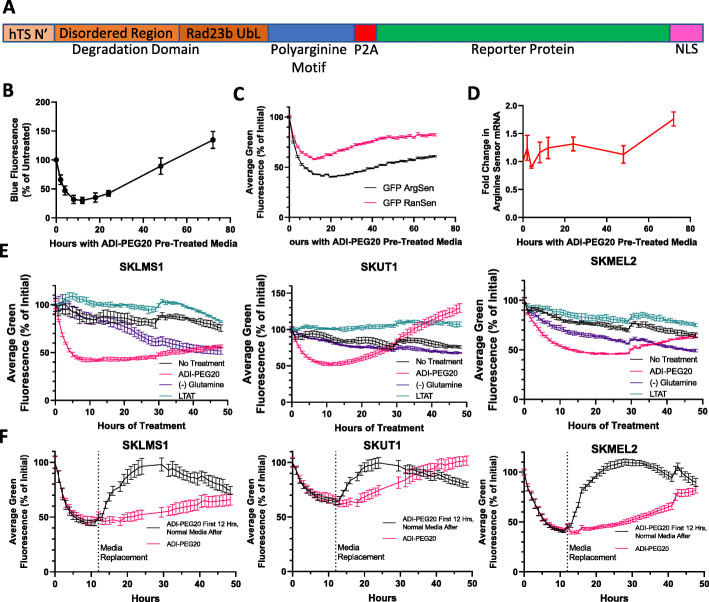
Design and demonstration of arginine sensor. **a** Diagram of the main components of the arginine sensor. **b** ArgSen fluorescence in SKLMS1 cells measured by flow cytometry. Error bars represent standard deviation. **c** GFP ArgSen and GFP RanSen fluorescence in SKLMS1 cells measured by microscopy. Error bars represent standard deviation. **d** Arginine sensor mRNA in SKLMS1 GFP ArgSen cells. Error bars represent standard error of the mean. **e** GFP ArgSen fluorescence with no treatment, glutamine deprivation, ADI-PEG20 treatment, and ADI-PEG20 resistance in three cell lines. Error bars represent standard deviation. **f** GFP ArgSen fluorescence with ADI-PEG20 treatment for 12 h, followed by normal media or continuation of treatment, in three cell lines. Error bars represent standard deviation

To gain a better understanding of the temporal emergence of resistance to ADI-PEG20 in real time, a florescence-based protein sensor was designed to measure arginine-dependent protein translation. A lentiviral delivery system is utilized to integrate the sensor gene into the host genome. The EF1α promoter was chosen to mimic endogenous arginine-dependent translation, as this drives homogeneous and stable transcription in human cells [29]. In addition, seven components were incorporated into the sensor (Fig. 2a). A fluorescent timer protein was used to optimize the first-generation reporter (ArgSen), as classical fluorescent proteins do not degrade fast enough for their fluorescence to noticeably decrease within a few hours of stopping translation. Fast-FT was chosen, as it matures from blue to red over a few hours in the cell, allowing only recently translated proteins to be measured by blue fluorescence [30]. However, most experiments were performed with an enhanced green fluorescent protein version of the sensor (GFP ArgSen), made possible by attaching a domain that rapidly degrades the GFP. Finally, a nuclear localization signal was added to enable better quantification via microscopy.

The core part of the arginine sensor is a polyarginine region that enables a more specific response to intracellular arginine levels (Fig. 2a). When the cell is starved of arginine with ADI-PEG20, this region should be translated more slowly, causing ribosomal pausing and/or stalling [24], resulting in slower translation of the downstream reporter protein. Twenty arginine residues are included in this region.

Upstream of and attached to the polyarginine motif is a strong proteasomal degradation signal (Fig. 2a), allowing the arginines to be recycled quickly, as the incorporated arginine residues might deplete cytoplasmic levels of the amino acid in the cell. The degradation domain also causes rapid depletion of the reporter when translation rates decrease, which is critical for the temporal sensitivity of the sensor. The degradation domain consists of three parts, Rad23b ubiquitin-like domain (UbL), the N terminus of human thymidylate synthase (hTS), and a disordered region. To promote ubiquitin-independent proteasomal degradation, a human Rad23b UbL domain was added. This domain has been shown to localize attached proteins to the proteasome and facilitate their rapid degradation [31]. hTS is degraded in a manner totally independent of ubiquitin [32]. The 30 amino acids from the N terminus of hTS are essential for its degradation and were therefore chosen as the N terminus of the sensor [32]. The hTS N terminus is short, but studies have shown that longer disordered regions generally lead to faster proteasomal degradation [33, 34]. For this reason, a long, disordered region was included. The measles virus phosphoprotein C-terminal domain flexible disordered linker was chosen as the long disordered region from a data set used to validate the Multilayered Fusion-based Disorder predictor v. 2.00 (MFDp2) [35–37]. Additional groups have confirmed that this region is a natural long, disordered linker [38–40].

Due to the intrinsic ability of Fast-FT to signal only recently translated proteins by blue fluorescence, a P2A site was included after the polyarginine motif and before the Fast-FT in this first version of the sensor. The P2A essentially acts as a self-cleavage site, and its inclusion is an effective way to translate two polypeptides from the same mRNA in the same quantity [41], which frees Fast-FT to function independently while recycling the upstream arginine (Fig. 2a). The GFP ArgSen, standardly used for most of the studies, does not include the P2A site, causing the GFP to be degraded quickly.

### Sensor response to ADI-PEG20

Flow cytometric analysis was performed on SKLMS1 cells expressing the Fast-FT arginine sensor (ArgSen) to characterize its fluorescence over time when cells were treated with ADI-PEG20 to deplete extracellular arginine. Flow cytometric analysis shows a decrease of ArgSen fluorescence with a minimum signal seen at 12 h, followed by a recovery of fluorescence back to untreated levels by 72 h (Fig. 2b), which is consistent with re-expression of ASS1 (Fig. 1b) and the reinitiation of proliferation (Fig. 1c). After transducing cells with GFP ArgSen, the response to ADI-PEG20 was compared to a variant in which the arginine residues of the polyarginine motif were replaced with random non-arginine amino acid residues (GFP RanSen) (Fig. 2c). This decreased the overall arginine content in the protein from 37 in GFP ArgSen to 17 residues in GFP RanSen, and the response to ADI-PEG20 was reduced correspondingly (Fig. 2c). GFP ArgSen mRNA levels do not decrease with ADI-PEG20 treatment, staying relatively steady for 48 h, with some increase but no decrease in transcription seen after that time, suggesting that this is a translational and not transcriptional probe for arginine starvation response (Fig. 2d).

Without treatment, sensor expression gradually decreases over time as cells grow, deplete nutrients, and become more confluent (Fig. 2e). In WT cells treated with ADI-PEG20, sensor fluorescence is reduced initially, then recovers over time, whereas untreated and LTAT cells maintain relatively steady fluorescence (Fig. 2e). Some cell lines, such as SKLMS1, are dependent on glutamine and therefore grow slowly in response to glutamine deprivation [28]. In response to glutamine withdrawal, SKLMS1 cells reduce GFP ArgSen signal overtime, but with slower kinetics than ADI-PEG20 treatment, and SKUT1 and SKMEL2 respond similarly (Fig. 2e). This is consistent with the sensor also responding to general protein translation. Glutamine deprivation has less effect on global translation rates than ADI-PEG20 in SKLMS1 (shown in Additional file 4), but Fig. 2c, e taken together indicate that GFP ArgSen expression is preferentially affected by arginine availability over other amino acids.

Cells were then treated with ADI-PEG20 for 12 h, near the minimum point of GFP ArgSen fluorescence, and then placed in normal growth media (Fig. 2f). GFP ArgSen fluorescence increased rapidly over the few hours following addition of complete media, stabilizing at roughly the baseline level, then slowly decreasing at a similar rate as untreated cells.

To test whether ASS1 expression is required for ADI-PEG20 resistance, GFP ArgSen was expressed in MEFs with *ASS1* either knocked out (*ASS1*^-/-^) or expressed at normal levels (*ASS1*^F/F^). When treated with ADI-PEG20, *ASS1*^F/F^ MEFs experienced a moderate decrease in fluorescence followed by recovery, whereas fluorescence decreased more rapidly and did not recover in *ASS1*^-/-^ MEFs (shown in Additional file 5). ADI-PEG20 also completely stopped the growth of *ASS1*^-/-^ MEFs but not *ASS1*^F/F^ MEFs (shown in Additional file 5).

### Sensor component characterization

**Fig. 3:**
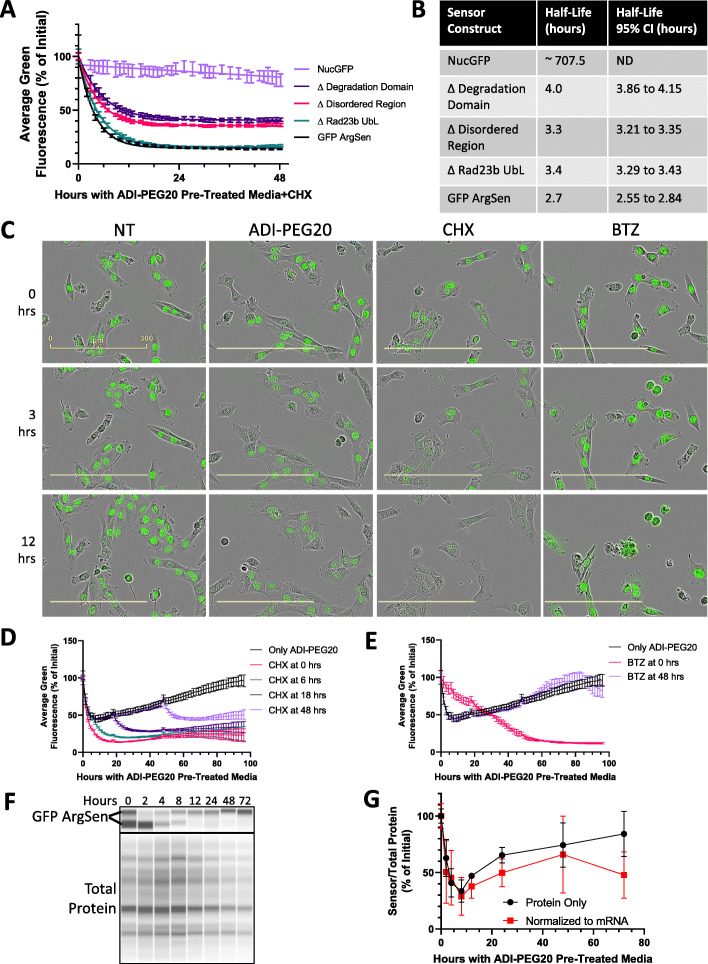
Validating degradation of sensor. **a** Degradation of GFP ArgSen and deletion variants in SKLMS1 in the presence of a translation inhibitor (CHX). Error bars represent standard deviation. **b** Best approximations of half-lives with 95% confidence intervals calculated from data in **a**. ND = not determined. **c** Representative images of SKLMS1 GFP ArgSen fluorescence during treatment with ADI-PEG20, CHX, or a proteasome inhibitor (BTZ). **d** GFP ArgSen fluorescence in SKLMS1 cells with ADI-PEG20, adding CHX at various time points. Error bars represent standard deviation. **e** GFP ArgSen fluorescence in SKLMS1 cells with ADI-PEG20, adding BTZ at various time points. Error bars represent standard deviation. **f** Immunoblots of GFP ArgSen protein in SKLMS1 over ADI-PEG20 time course. **g** Quantification of **f**, along with the same data normalized to corresponding mRNA levels from 2 days. Error bars represent standard error of the mean

Degradation and recycling of the sensor probe’s arginines are important for temporal resolution and to avoid unwanted arginine depletion. The GFP ArgSen is in a constant state of balance between rapid synthesis and rapid degradation, resulting in a relatively low steady state level of GFP due to the incorporation of its rapid degradation domain. Any disturbance of either translation or degradation, such as ADI-PEG20 treatment or translation or proteasome inhibitors, should affect the levels of GFP in the cell. With this design, faster degradation gives better temporal resolution to the sensor. For this reason, we characterized the importance of each upstream (of reporter) sensor component to the efficiency of degradation. GFP ArgSen degradation was measured in the presence of the translation inhibitor cycloheximide (CHX), and this was compared to variants with deletions of various regions upstream of the GFP (Fig. 3a). The complete sensor (GFP ArgSen) was found to degrade the fastest, as measured by its half-life of approximately 2.7 h (Fig. 3b). However, deletion of either the designed disordered region or the ubiquitin-like domain led to only a slight increase in half-life of less than 1 h (Fig. 3b). All variants that included the polyarginine motif degraded much faster than nuclear GFP alone (labeled NucGFP; > 100 h half-life), including the variant with no degradation domain (Fig. 3b).

ADI-PEG20 causes a similar but slower decline in sensor fluorescence when compared to CHX (Fig. 3c). Images show that translation inhibitor CHX rapidly depletes sensor fluorescence in cells, while proteasomal inhibitor bortezomib (BTZ) temporarily increases fluorescence before cell death (Fig. 3c). To test whether translation and proteasomal degradation of the sensor continue even after arginine depletion, SKLMS1 WT cells were treated with CHX at multiple time points after starting ADI-PEG20 treatment. Results demonstrated the expected pattern of a rapid decrease in fluorescence, indicating that proteasomal degradation is not significantly affected by ADI-PEG20 (Fig. 3d). Next, cells were treated with BTZ at 0 or 48 h after ADI-PEG20 treatment. BTZ treatment at either time point resulted in higher sensor fluorescence in the following hours compared to control (Fig. 3e).

To correlate sensor fluorescence with protein expression by an independent method, immunoblots by capillary electrophoresis for arginine sensor reporter protein were performed over a time course of ADI-PEG20 in SKLMS1 WT, showing a similar kinetic pattern as flow cytometry and microscopy measurements (Fig. 3f, g). The pattern of sensor expression is also relatively unchanged when normalized to mRNA levels, with the exception of a moderate decrease at 72 h due to increased mRNA at that time (Fig. 3g).

### Single cell tracking

**Fig. 4:**
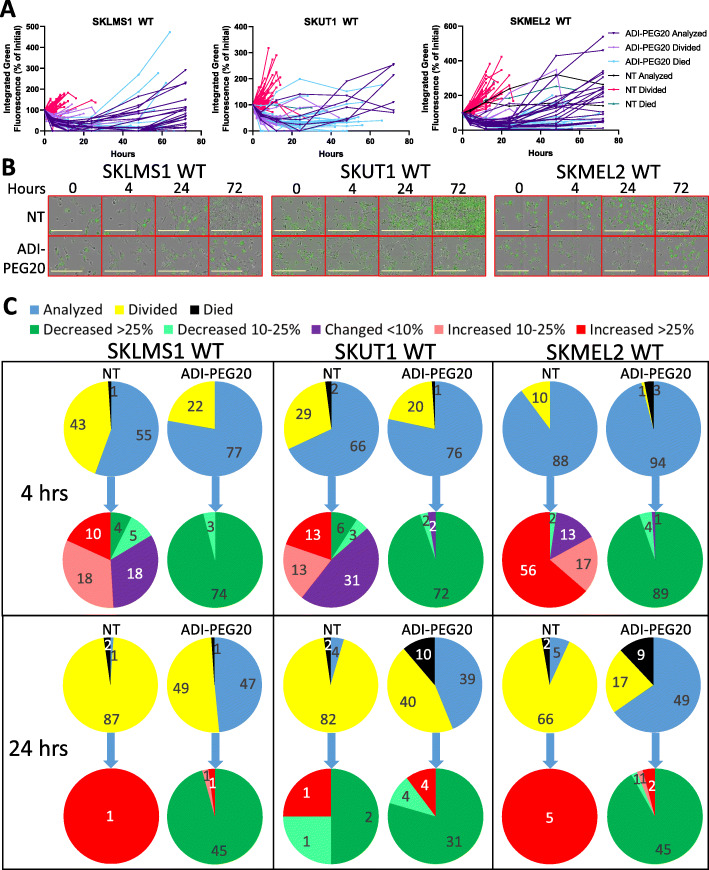
Individual cell responses to arginine deprivation. **a** GFP ArgSen fluorescence in individual cells over time in three cell lines. **b** Representative images of growth and GFP ArgSen fluorescence over 3 days with or without ADI-PEG20 in three cell lines. **c** Counts of cell fates and tiered changes in GFP ArgSen fluorescence at 4 and 24 h with and without ADI-PEG20 in three cell lines

To determine the dynamics of cellular responses to arginine deprivation, integrated green fluorescence intensities of individual WT cells were tracked over time with and without arginine depletion by ADI-PEG20. In the absence of arginine depletion, the majority of cells increased or maintained relatively steady sensor fluorescence before undergoing mitosis (Fig. 4a). With ADI-PEG20 treatment, every measured cell decreased expression of the sensor before some cells recovered expression over time (Fig. 4a). The timing of recovery varied among cells, and the magnitude varied even more, indicating heterogeneous resistance (Fig. 4a). Higher proportions of cells in Fig. 4a die with ADI-PEG20 treatment than are shown Fig. 1d. This is because Fig. 4a does not include daughter cells from divisions, which increase the total live cell numbers by 72 h.

SKLMS1, SKUT1, and SKMEL2 WT cells with and without ADI-PEG20 over time were imaged with the IncuCyte S3 (Fig. 4b). Representative images at 4, 24, and 72 h are shown. At 4 and 24 h, most treated cells have significantly decreased fluorescence compared to their untreated controls. This fluorescence is recovered in some treated cells at 72 h, while untreated cells have become confluent, causing downregulation of translation, especially in SKLMS1 and SKMEL2.

We then determined the percentage of cells that had divided, died or neither (labeled as “analyzed”) at both 4 and 24 h, shown in Fig. 4c. In each cell line at both time points, a significantly lower percentage of cells treated with ADI-PEG20 had divided, supporting the growth data in Fig. 1c. The cells which neither divided nor died through each time point were then categorized according to their change in arginine sensor fluorescence. At 4 h, no treated cells had increased expression of the sensor, while over 83% of untreated cells in each cell line had increased expression or had not changed. At 24 h, fluorescence remained low in 90–96% of treated cells, while the remaining subset had already started to recover (Fig. 4a, c). Less than 7% of untreated cells in any cell line neither divided nor died by 24 h, limiting the usefulness of categorizing. A subset of cells that divided and their descendants were able to be tracked, but most descendants were lost due to confluent crowding and imaging limitations of the IncuCyte S3. Those descendants that could be tracked with confidence showed a very similar pattern of sensor fluorescence as cells that did not divide within 72 h (shown in Additional file 6).

### Metabolic analysis

**Fig. 5:**
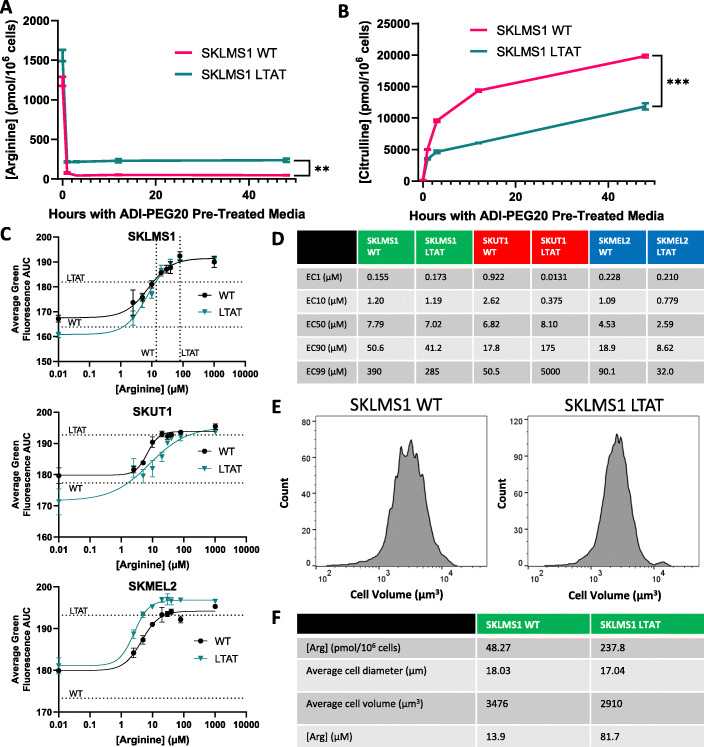
Effects of intracellular arginine concentrations on translation. **a** Arginine concentrations in SKLMS1 WT and LTAT cells expressing GFP ArgSen over time with ADI-PEG20 treatment as measured by mass spectrometry. **b** Citrulline concentrations in SKLMS1 WT and LTAT cells expressing GFP ArgSen over time with ADI-PEG20 treatment. **c** Average GFP ArgSen fluorescence over 2 h with various concentrations of arginine in media in three cell lines. Horizontal dotted lines indicate the values with ADI-PEG20 treatment. Vertical dotted lines indicate calculated intracellular arginine concentrations after 48 h of ADI-PEG20 treatment. **d** Effective concentration (EC) values for arginine driving translation of GFP ArgSen in three cell lines, calculated from **c**. **e** Histograms of cell volumes after 48 h of ADI-PEG20 treatment. **f** Intracellular arginine concentrations after 48 h of ADI-PEG20 treatment, along with values used to calculate concentrations. All error bars represent standard deviation

Metabolomic analysis by mass spectrometry of SKLMS1 WT and LTAT cells treated with ADI-PEG20 over time demonstrates a decrease in intracellular arginine concentration that occurs within 1 h (Fig. 5a). Arginine levels do not recover within 48 h (Fig. 5a). However, the steady state intracellular arginine levels are roughly five times higher in LTAT cells than in WT cells when treated with ADI-PEG20 (Fig. 5a). As expected, intracellular citrulline levels start low and increase greatly over time (Fig. 5b). Maximum citrulline levels are not reached by 12 h, and citrulline levels stay significantly lower in LTAT cells than in WT cells (Fig. 5b), as expected in cells with a functional amount of ASS1.

### Minimum arginine concentration needed for optimal sensor translation

In order to determine concentrations of arginine needed for optimal sensor translation, WT and LTATs of each cell line were treated with media containing varying concentrations of arginine, and the fluorescence of GFP ArgSen was quantified by microscopy (Fig. 5c). These conditions limit the arginine concentrations of both WT and LTAT cells, as the media contains no citrulline that could be used by ASS1 to synthesize arginine, and no cells outside of the small intestine express all the necessary genes to synthesize citrulline de novo [42]. EC50s were found to range from 2.59 to 8.10 μM (Fig. 5d). To compare arginine sensor translation at various arginine media concentrations to actual intracellular arginine concentrations with ADI-PEG20, a combination of microscopy and flow cytometry was used to measure the diameters and calculate the volumes of individual cells. Then, intracellular concentrations of arginine were calculated using metabolomics data and average cell volumes at 48 h (Fig. 5e, f). The resulting figures were 13.9 μM of intracellular arginine for WT cells and 81.7 μM for LTAT cells.

## Discussion

The ArgSen is a new single-cell sensor to monitor real-time arginine-dependent protein translation. Arginine starvation strategies are being developed clinically, but a single agent use of therapeutics like ADI-PEG20 has failed to translate to patients [43]. Therefore, an understanding of the cellular adaption to arginine deprivation is an unmet need. By understanding the intracellular dynamics of arginine starvation, a better understanding of when to add additional agents may lead to better therapeutic strategies. Therefore, the development of the ArgSen will allow not only for an understating of the temporal dynamics of arginine starvation, but also the stromal response when multiple colored sensors are fully implemented in vitro and in vivo, which is the subject of ongoing work.

The specificity of the ArgSen to arginine is supported by two experiments. First, the initial response to arginine deprivation is greatly blunted when arginine residues in the polyarginine motif are replaced by random non-arginine amino acids (RanSen). Second, glutamine deprivation, while decreasing the maximal rate of translation, slows synthesis much more gradually than arginine deprivation. These results indicate a preference and specificity of the sensor for monitoring arginine-rich translation and support the idea that the dominant mechanism of regulation is a decrease in translation. For use in other systems where arginine deficiency may or may not be occurring, the RanSen may be used as a control for ArgSen to determine whether the observed response is preferential for arginine availability.

The ArgSen demonstrates a rapid decrease in expression followed by a gradual recovery over time in ASS1-deficient cells. The Fast-FT ArgSen and GFP ArgSen show some differences in the magnitude of response to ADI-PEG20. This is likely attributable to the different mechanisms by which their signals are degraded, as well as different methods of measurement, as average GFP ArgSen fluorescence is susceptible to overestimation, as some cells fall below the detection threshold.

In contrast to WT cells, resistant LTAT cells demonstrate no change in expression in response to ADI-PEG20, as expected. This long-term resistance is conferred mostly by increased ASS1 expression. WT cells also show a short-term increase in ASS1 corresponding to ArgSen recovery from ADI-PEG20 treatment. However, this increase in ASS1 expression likely does not totally account for the increased sensor expression, as autophagy is known to help these cells cope with arginine starvation. Post-translational ASS1 activity regulation also cannot be ruled out. The sensor is a more dynamic measure of resistance, as it increases significantly by 24 h, whereas ASS1 increases are small and difficult to measure at this early time point. Both continue to increase thereafter.

The ArgSen model depends on constant sensor mRNA levels in the cell for reporter protein levels to accurately reflect the ability of the cell to use arginine for translation. When measured over a time course of ADI-PEG20 treatment, sensor mRNA levels do not significantly decrease at any point, and the overall pattern of sensor fluorescence is very similar regardless of whether it is normalized to mRNA, confirming that sensor protein expression levels are driven by translation rather than transcription.

Next, while the full-length sensor possesses the shortest half-life and therefore the best temporal resolution, deletion of some parts of the degradation domain still resulted in only marginally longer half-lives, specifically when the polyarginine motif remained. We speculate that this is because the polyarginine motif is likely disordered, and the presence of a disordered region is critical and often sufficient for proteasomal degradation [32, 44, 45].

Finally, CHX and BTZ worked as expected in sensor-expressing cells, rapidly depleting or causing a buildup of the reporter protein, respectively. This supports the proposed mechanism of the sensor that relies on both rapid synthesis and rapid degradation, leading to a relatively low steady-state level of reporter protein. Immunoblots further confirmed that the reporter protein is indeed being degraded as designed.

Tracking of arginine sensor expression in individual ASS1-deficient cells demonstrated that ADI-PEG20 treatment initially depleted the reporter protein in every cell analyzed, without exception. This is strong evidence that resistant cells are not present in naïve populations. Subsequent recovery of expression indicates a metabolic adaptation in response to arginine deprivation, but the magnitude of recovery varies widely. While population averages show a slow recovery of expression back to roughly untreated levels by 72 h, depending on the cell line, single-cell tracking reveals a diverse range of responses. After 3 days, many cells still express very little arginine sensor, while some have roughly normal levels, and a few even increased well past initial levels. The timing of recovery also varies among cells, with a small subset gaining resistance earlier than most. These cells are present in all three cell lines, but they are most prominent in SKUT1. Throughout the study, SKUT1 seemed to recover expression more quickly than the other cell lines, which agrees with the fact that it has a higher basal level of ASS1 expression [28]. These results overall show a fairly homogeneous initial response to ADI-PEG20 followed by a heterogeneous recovery period wherein some cells increase expression while others remain low.

When EC50s of arginine for sensor translation were measured, no difference between WT and LTAT of the same cell line exceeded 2 μM, as LTAT cells are able to increase their supply of arginine during ADI-PEG20 treatment rather than using it more efficiently. Measurements of actual arginine concentrations in cells with ADI-PEG20 treatment suggest that LTATs are able to maintain their concentrations of intracellular arginine at roughly five times the level of WT cells. While SKLMS1 LTAT arginine levels are still far below those of untreated media (about 340 μM, shown in Additional file 7), the cells maintain a concentration that is nearly twice their EC90, explaining why translation in LTATs is unaffected by ADI-PEG20. However, the intracellular concentration of arginine in WT cells is substantially below this level when treated with ADI-PEG20, which greatly inhibits protein translation.

Interestingly, after the start of treatment with ADI-PEG20, citrulline takes many hours to reach a steady intracellular concentration. This is likely because these cells do not readily transport citrulline across the plasma membrane. WT cells maintain higher citrulline levels than LTATs, likely as a result of faster conversion to arginine by ASS1 in LTATs. More importantly, and unexpectedly, arginine levels did not significantly recover within 48 h of ADI-PEG20 treatment. Pairing this with the previous data, arginine sensor expression is seen to increase in WT cells while intracellular arginine concentrations remain constant. This seems contradictory, but the most likely mechanism is that, after initial arginine depletion, synthesis of arginine increases over time, and utilization of arginine increases to match, thereby maintaining a steady concentration of arginine while increasing translation. We performed experiments to test this hypothesis by inhibiting both translation and proteasomal degradation separately up to 48 h after the initiation of ADI-PEG20 treatment. The results showed that robust translation and degradation continue throughout the course of arginine deprivation and that the sensor we have designed measures adaptive resistance more accurately than actual intracellular arginine concentrations by directly monitoring the ability of cells to use arginine for translation.

## Conclusions

The ability to track responses in individual cells makes the sensor useful for the study of arginine deprivation for clinical development. Using the sensor, multiple ASS1-deficient cell lines were shown to completely lack resistance to ADI-PEG20 at the single-cell level in naïve populations but develop a heterogeneous pattern of resistance. This is promising for the potential effectiveness of treatment with ADI-PEG20 and combination therapies with it. The finding that resistance is heterogeneous suggests that temporally targeting the early response to ADI-PEG20 with additional drugs may yield a more effective, homogeneous response. Finally, the concept of the sensor could be applied to other amino acids through modification, having potential to be used in the study of many other biological systems.

## Supplementary Information

**Additional file 1: Table S1.** List of cell lines and sources.

**Additional file 2: Table S2.** List of antibodies and sources.

**Additional file 3: Table S3.** List and full sequences of DNA oligonucleotides used in this study.

**Additional file 4: Figure S1.** Global protein translation rates in SKLMS1 WT over 12 hours with no treatment, ADI-PEG20, glutamine deprivation, or ADI-PEG20 plus glutamine deprivation. Error bars represent standard deviation.

**Additional file 5: Figure S2.** GFP ArgSen fluorescence and growth over 72 hours with ADI-PEG20 treatment in *ASS1*^F/F^ and *ASS1*^-/-^ MEFs. Error bars represent standard deviation.

**Additional file 6: Figure S3.** Analysis of GFP ArgSen fluorescence in subsets of dividing cells from Figure 4. Five untreated cells and roughly 20 ADI-PEG20-treated cells from each cell type were tracked, along with all their descendants, for 24 and 72 hours respectively, excluding cells that could not be tracked with confidence. Data points represent the average green fluorescence of cells at the indicated timepoint, calculated from the averages of each initial cell or its trackable descendants at that time. Error bars represent standard error of the mean.

**Additional file 7: Figure S4.** Concentrations of arginine in MEM after 72 hours with SKLMS1 WT and LTAT cells with and without ADI-PEG20 treatment. Bars for ADI-PEG20-treated media are not visible because values are too low. Error bars represent standard deviation.

## Data Availability

The datasets used and/or analyzed during the current study are available from the corresponding author on reasonable request.

## Abbreviations

ADI-PEG20: PEGylated arginine deiminase
ASS1: Argininosuccinate synthetase 1
BTZ: Bortezomib
cDNA: Complementary deoxyribonucleic acid
CHX: Cycloheximide
DNA: Deoxyribonucleic acid
EC: Effective concentration
EF1α: Elongation factor 1 alpha
EUCOMM: European Conditional Mouse Mutagenesis Program
FBS: Fetal bovine serum
hTS: Human thymidylate synthase
GFP: Enhanced green fluorescent protein
IMDM: Iscove’s modified Dulbecco’s medium
LTAT: Long-term ADI-PEG20-treated
MEF: Mouse embryonic fibroblast
MEM: Minimum Essential Media
MFDp2: Multilayered Fusion-based Disorder predictor v. 2.00
mRNA: Messenger ribonucleic acid
mTOR: Mammalian target of rapamycin
NLS: Nuclear localization signal
PBS: Phosphate-buffered saline
PCR: Polymerase chain reaction
RNA: Ribonucleic acid
RT-qPCR: Quantitative reverse transcription PCR
RPMI: Roswell Park Memorial Institute
SILAC: Stable Isotope Labeling by/with Amino acids in Cell culture
SV40: Simian virus 40
WT: Wild type
UbL: Ubiquitin-like
