# Supplementary material for: Intracellular arginine-dependent translation sensor reveals the dynamics of arginine starvation response and resistance in ASS1-negative cells
Source: Cancer Metab. 2021 Jan 21;9:4. doi: 10.1186/s40170-021-00238-9 (PMC7818940; doi:10.1186/s40170-021-00238-9)
Supplement: Supplementary file 1 — Additional file 1: Table S1. List of cell lines and sources. [file 40170_2021_238_MOESM1_ESM.docx]

**Table S1: Cell Lines**

| **Cell Line** | **Vendor** | **Catalog Number** |
| --- | --- | --- |
| SKLMS1 | ATCC | HTB-88 |
| SKUT1 | ATCC | HTB-114 |
| SKMEL2 | ATCC | HTB-68 |
