# Supplementary material for: Intracellular arginine-dependent translation sensor reveals the dynamics of arginine starvation response and resistance in ASS1-negative cells
Source: Cancer Metab. 2021 Jan 21;9:4. doi: 10.1186/s40170-021-00238-9 (PMC7818940; doi:10.1186/s40170-021-00238-9)
Supplement: Supplementary file 2 — Additional file 2: Table S2. List of antibodies and sources. [file 40170_2021_238_MOESM2_ESM.docx]

**Table S2: Antibodies**

| **Antibody** | **Vendor** | **Catalog Number** |
| --- | --- | --- |
| ASS1 | Polaris | - |
| ASS1 | Abcam | ab175607 |
| GFP | Santa Cruz Biotechnology | sc-9996 |
