# Supplementary material for: Intracellular arginine-dependent translation sensor reveals the dynamics of arginine starvation response and resistance in ASS1-negative cells
Source: Cancer Metab. 2021 Jan 21;9:4. doi: 10.1186/s40170-021-00238-9 (PMC7818940; doi:10.1186/s40170-021-00238-9)
Supplement: Supplementary file 3 — Additional file 3: Table S3. List and full sequences of DNA oligonucleotides used in this study. [file 40170_2021_238_MOESM3_ESM.docx]

**Table S3: Oligonucleotides**

| **Name** | **Sequence** |
| --- | --- |
| EF1a fwd | TCAAGCCTCAGACAGTGGTTC |
| pKLV2 seq rev | CATAGCGTAAAAGGAGCAAC |
| pLV seq rev | CTTCGGCCAGTAACGTTAGG |
| Rad23b end fwd | GGTGACAAAGCCGAAAGC |
| SalI BamHI FastFT fwd | GACGTGGAGGAGAACCCTGGACCTGTCGACGGATCCATGAGTAAGGGCGAGGAGGATAAC |
| NotI NLS FastFT rev | ATCCAGAGGTTGAGCGGCCGCTTATACCTTACGCTTCTTCTTTGGCTTGTAAAGCTCATC |
| SalI BamHI GFP fwd | GACGTGGAGGAGAACCCTGGACCTGTCGACGGATCCATGGTGAGCAAGGGCGAGGAGCTG |
| NotI NLS GFP rev | ATCCAGAGGTTGAGCGGCCGCTTATACCTTACGCTTCTTCTTTGGCTTGTACAGCTCGTC |
| Proteasomal del fwd | CGCGCCCACCATGGCGTT |
| Proteasomal del rev | CGAACGCCATGGTGGG |
| Disordered del fwd | CGCGCCCACCATGGCGGGTATGCA |
| Disordered del rev | TACCCGCCATGGTGGG |
| Rad23b del fwd | TGCTAGCGGATTCGAAT |
| Rad23b del rev | CTAGATTCGAATCCGCTAGCATGCA |
| Reporter only fwd | CGCGCCCACCATGGCGG |
| Reporter only rev | GATCCCGCCATGGTGGG |
| qrtPCR GAPDH fwd | CGGATTTGGTCGTATTGGGC |
| qrtPCR GAPDH rev | CCGTTCTCAGCCATGTAGTTG |
| qrtPCR ArgSen fwd | CGAACGTCTTCAAGGGGACA |
| qrtPCR ArgSen rev | GTATCGGGCACAAAGCCAAC |
