# Supplementary figures and images for: Intracellular arginine-dependent translation sensor reveals the dynamics of arginine starvation response and resistance in ASS1-negative cells
Source: Cancer Metab. 2021 Jan 21;9:4. doi: 10.1186/s40170-021-00238-9 (PMC7818940; doi:10.1186/s40170-021-00238-9)

## Slide 1
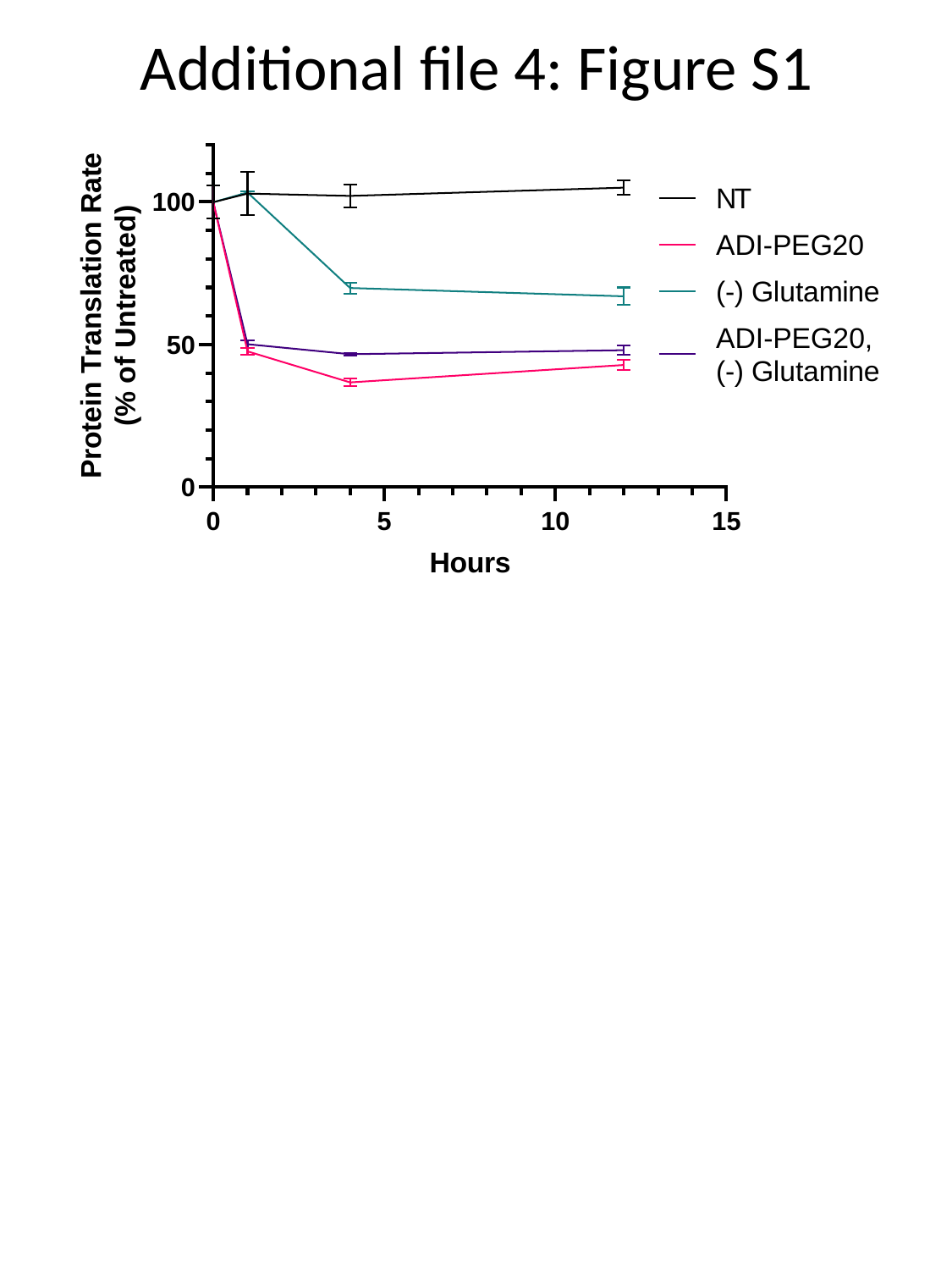

Additional file 4: Figure S1

Supplement: Supplementary file 4 — Additional file 4: Figure S1. Global protein translation rates in SKLMS1 WT over 12 hours with no treatment, ADI-PEG20, glutamine deprivation, or ADI-PEG20 plus glutamine deprivation. Error bars represent standard deviation. [file 40170_2021_238_MOESM4_ESM.pptx]

## Slide 1
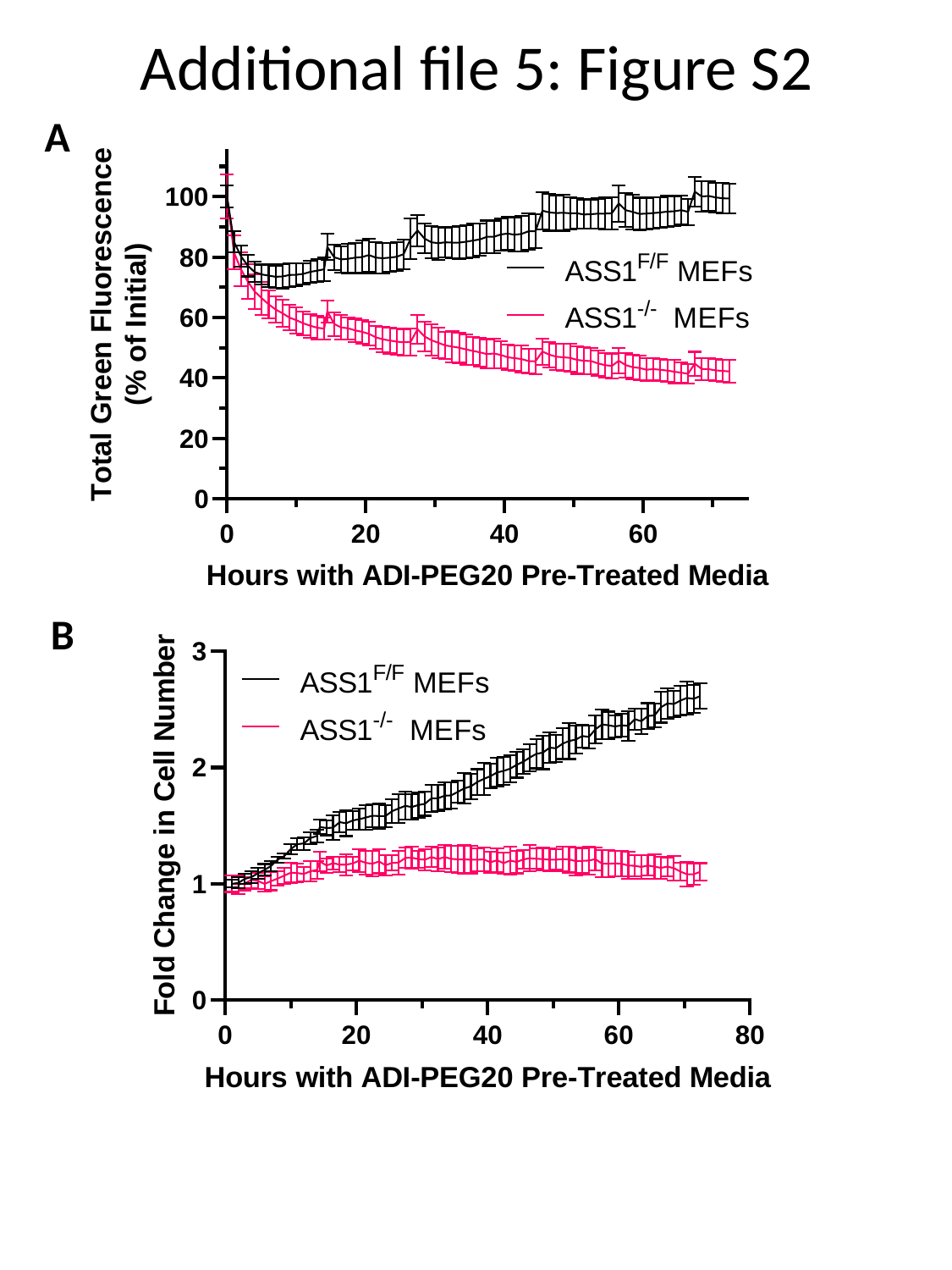

Additional file 5: Figure S2
A
B

Supplement: Supplementary file 5 — Additional file 5: Figure S2. GFP ArgSen fluorescence and growth over 72 hours with ADI-PEG20 treatment in ASS1F/F and ASS1-/- MEFs. Error bars represent standard deviation. [file 40170_2021_238_MOESM5_ESM.pptx]

## Slide 1
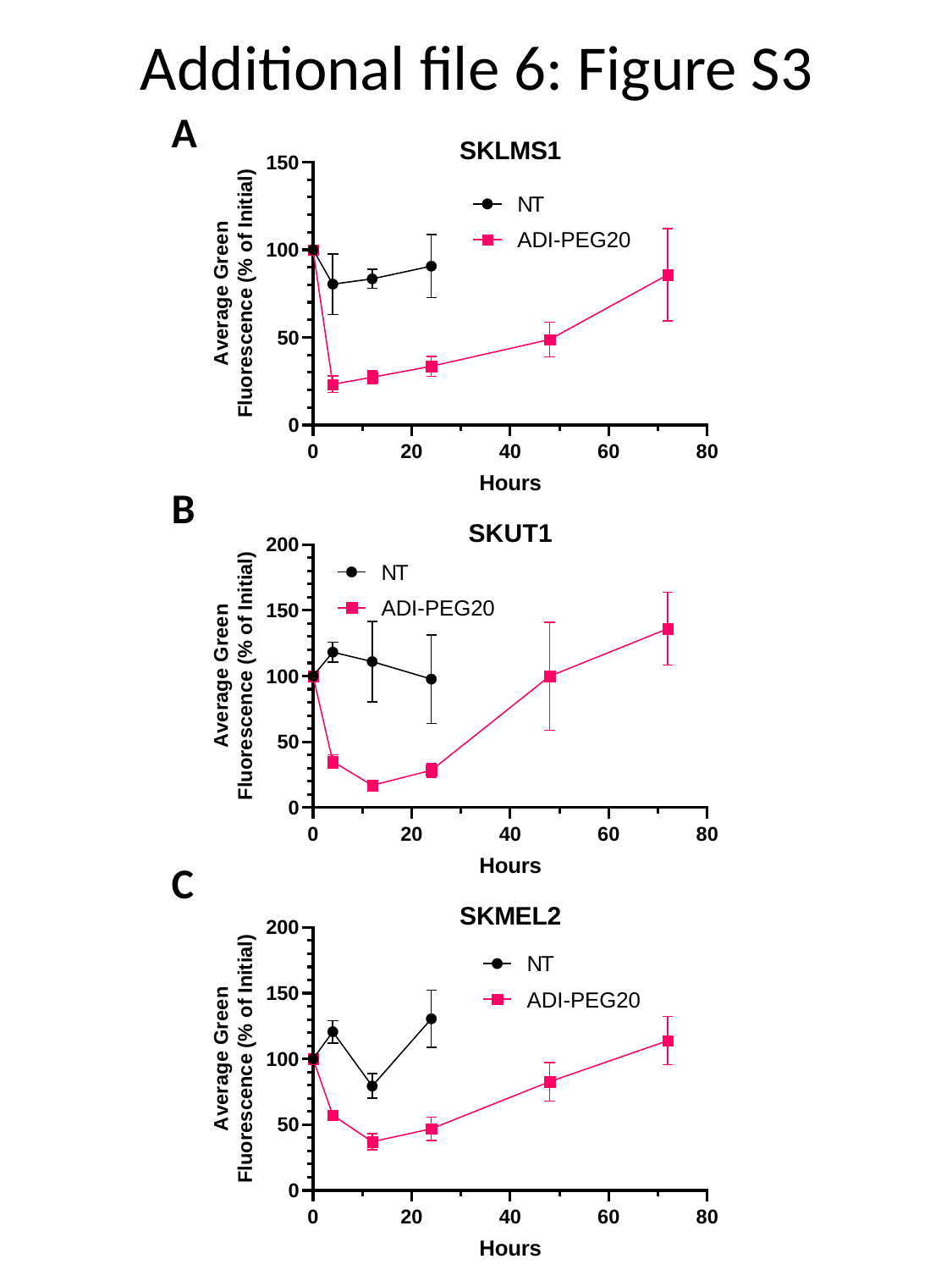

Additional file 6: Figure S3
A
B
C

Supplement: Supplementary file 6 — Additional file 6: Figure S3. Analysis of GFP ArgSen fluorescence in subsets of dividing cells from Figure 4. Five untreated cells and roughly 20 ADI-PEG20-treated cells from each cell type were tracked, along with all their descendants, for 24 and 72 hours respectively, excluding cells that could not be tracked with confidence. Data points represent the average green fluorescence of cells at the indicated timepoint, calculated from the averages of each initial cell or its trackable descendants at that time. Error bars represent standard error of the mean. [file 40170_2021_238_MOESM6_ESM.pptx]

## Slide 1
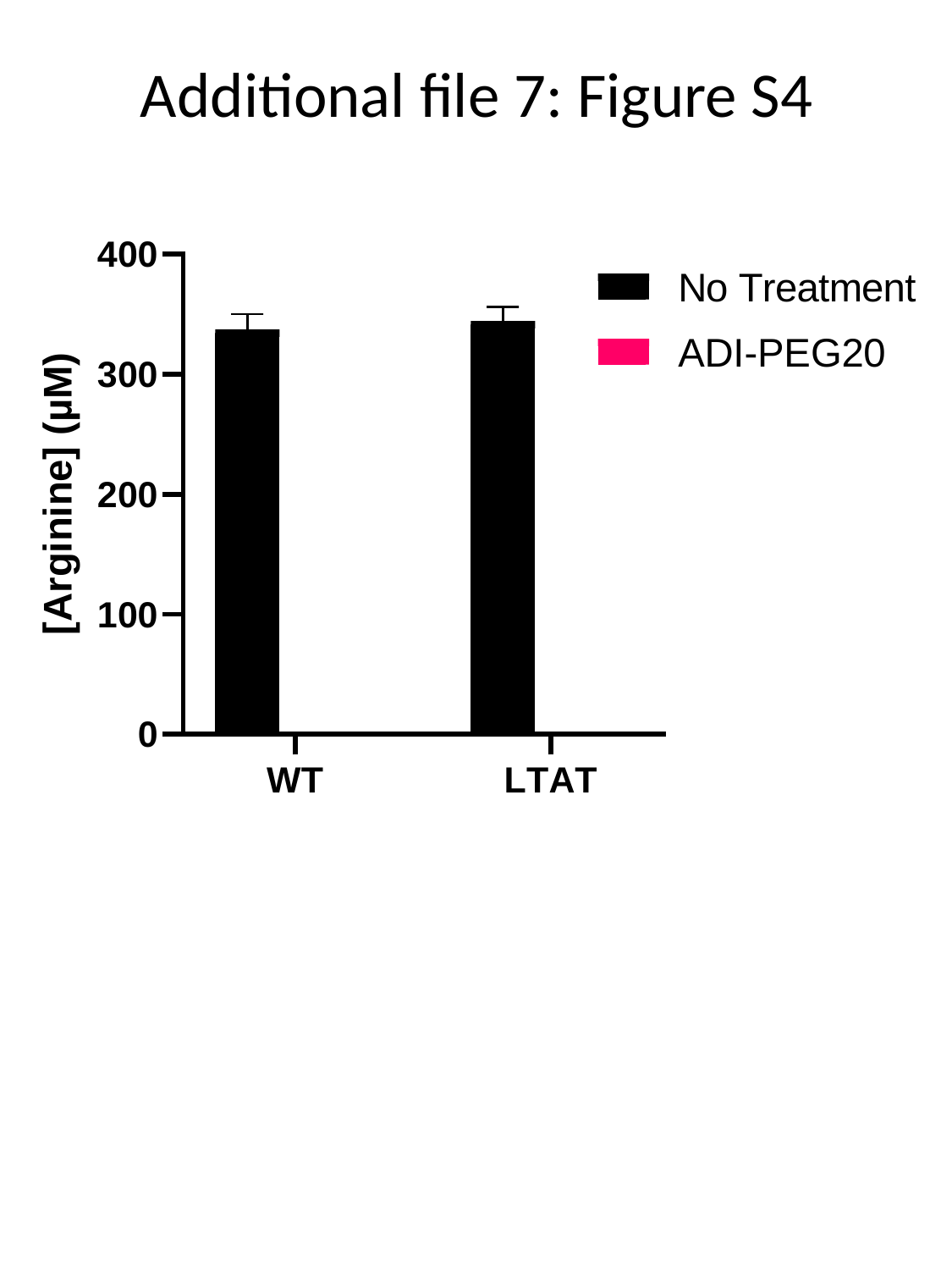

Additional file 7: Figure S4

Supplement: Supplementary file 7 — Additional file 7: Figure S4. Concentrations of arginine in MEM after 72 hours with SKLMS1 WT and LTAT cells with and without ADI-PEG20 treatment. Bars for ADI-PEG20-treated media are not visible because values are too low. Error bars represent standard deviation. [file 40170_2021_238_MOESM7_ESM.pptx]
